# ANGPTL4 is a potential driver of HCV-induced peripheral insulin resistance

**DOI:** 10.1038/s41598-023-33728-5

**Published:** 2023-04-25

**Authors:** Diana Gomes, Cyril Sobolewski, Stéphanie Conzelmann, Tifany Schaer, Etienne Lefai, Dulce Alfaiate, Eirini D. Tseligka, Nicolas Goossens, Caroline Tapparel, Francesco Negro, Michelangelo Foti, Sophie Clément

**Affiliations:** 1 Department of Pathology and Immunology, University of Geneva, Geneva, Switzerland; 2 Cell Physiology and Metabolism, University of Geneva, Geneva, Switzerland; 3 Unité de Nutrition Humaine, INRAE, Université Clermont Auvergne, 63000 Clermont-Ferrand, France; 4 Gastroenterology and Hepatology Division, University Hospitals, Geneva, Switzerland; 5 Department of Microbiology and Molecular Medicine, University of Geneva, Geneva, Switzerland; 6 Clinical Pathology Division, University Hospitals, Geneva, Switzerland; 7 Present Address: Koch Institute for Integrative Cancer Biology, Massachusetts Institute of Technology, Cambridge, MA USA; 8 Present Address: U1286-INFINITE-Institute for Translational Research in Inflammation, CHU Lille, Inserm, University Lille, 59000 Lille, France; 9 Present Address: Department of Infectious Diseases, Hôpital de la Croix Rousse, Lyon University Hospitals, Lyon, France

**Keywords:** Molecular medicine, Gastroenterology, Hepatology, Pathogenesis, Infection

## Abstract

Chronic hepatitis C (CHC) is associated with the development of metabolic disorders, including both hepatic and extra-hepatic insulin resistance (IR). Here, we aimed at identifying liver-derived factor(s) potentially inducing peripheral IR and uncovering the mechanisms whereby HCV can regulate the action of these factors. We found *ANGPTL4* (Angiopoietin Like 4) mRNA expression levels to positively correlate with HCV RNA (r = 0.46, *p* < 0.03) and HOMA-IR score (r = 0.51, *p* = 0.01) in liver biopsies of lean CHC patients. Moreover, we observed an upregulation of *ANGPTL4* expression in two models recapitulating HCV-induced peripheral IR, i.e. mice expressing core protein of HCV genotype 3a (HCV-3a core) in hepatocytes and hepatoma cells transduced with HCV-3a core. Treatment of differentiated myocytes with recombinant ANGPTL4 reduced insulin-induced Akt-Ser473 phosphorylation. In contrast, conditioned medium from *ANGPTL4*-KO hepatoma cells prevented muscle cells from HCV-3a core induced IR. Treatment of HCV-3a core expressing HepG2 cells with PPARγ antagonist resulted in a decrease of HCV-core induced *ANGPTL4* upregulation. Together, our data identified ANGPTL4 as a potential driver of HCV-induced IR and may provide working hypotheses aimed at understanding the pathogenesis of IR in the setting of other chronic liver disorders.

## Introduction

Hepatitis C virus (HCV) is a major cause of chronic liver disease, affecting an estimated 71 million people worldwide (~ 1% of the global population) and causing ~ 400,000 deaths annually^1,2^. Morbidity and mortality due to chronic hepatitis C (CHC) is not only associated with liver-related sequelae, including decompensated cirrhosis and hepatocellular carcinoma (HCC), but also with a vast range of extrahepatic manifestations, including cryoglobulinemic vasculitis, lymphoma, cardiovascular diseases, insulin resistance (IR) and type 2 diabetes (T2D)^3^.

The recent advent of potent antivirals sparked the implementation of national strategies for viral elimination by 2030^4^. Direct-acting antivirals (DAAs) display much greater safety and efficacy than the prior interferon-based regimens (> 95% vs. 50% cure rates, respectively)^5^, with significant implications for the management of both hepatic and extrahepatic sequelae of the viral infection. Recent international guidelines^6,7^ recommended the DAA treatment implementation without delay in patients with significant extrahepatic manifestations of HCV infection.

HCV infection significantly impairs the glucose metabolism, leading to IR and T2D. The association between HCV infection and T2D has been supported by several experimental and population-based studies^8–12^. HCV-induced IR contributes to a faster progression of the liver and extrahepatic diseases, leading to a worsening of the clinical scenario. IR is associated with accelerated liver fibrosis progression^13,14^, increased risk factor for malignancy^15,16^ and high cardiovascular mortality^17^. Clearance of HCV results in an improved IR that reduces the risk of developing T2D during follow-up and exerts beneficial effects on both liver- and non-liver-related outcomes^18–20^.

Albeit HCV mainly infects hepatocytes, euglycemic-hyperinsulinemic clamp measurements in CHC patients reported a reduced insulin-induced glucose uptake in extrahepatic sites—i.e. adipose tissue and skeletal muscles^21–23^. An extrahepatic component of IR in HCV infection may thus imply that the infected liver, through endocrine mechanisms, is able to modify the insulin sensitivity of peripheral uninfected sites. Indeed, a vast number of liver-derived endocrine factors have been associated with peripheral glucose metabolism alterations^24–27^. Here, we aimed at identifying liver-derived factor(s) involved in peripheral IR that could be potentially modulated by HCV. To this goal, we combined human studies with the development of a proof-of-concept cell culture-based system and the generation of an HCV mouse model, to analyze the crosstalk between the liver and peripheral organs. By using these models, we identified ANGPTL4, a circulating protein highly expressed in liver, to be potentially involved in HCV-driven peripheral IR.

## Results

### *ANGPTL4* expression in CHC patients correlates with levels of insulin resistance

**Figure 1:**
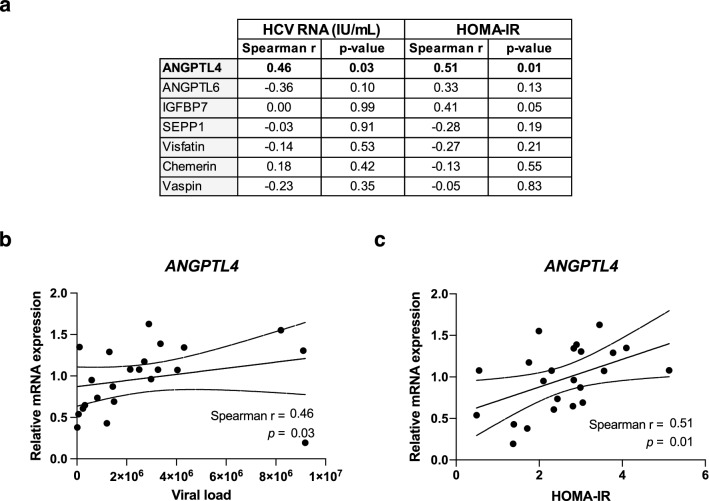
Hepatic mRNA expression of ANGPTL4 correlates with viral load and insulin resistance in CHC patients. (**a**) Correlation analysis between mRNA expression levels of ANGPTL-4 and -6, IGFBP7, SEPP1, visfatin, chemerin and vaspin with HCV RNA (IU/mL) and HOMA-IR in lean CHC patients (*n* = 23); (**b-c**) correlation between liver *ANGPTL4* mRNA expression and HCV RNA (B) and HOMA-IR (**c**).

In a previous study, we showed that viral clearance following an IFNα-free, antiviral therapy leads to an improved peripheral insulin sensitivity in lean, non-diabetic patients infected with HCV genotype 3, and that HCV modifies the circulating levels of metabolic active factors likely involved in the pathogenesis of peripheral IR. As an attempt to identify such factors, we have previously measured the plasma level of several hepatokines and other factors known to be involved in glucose and lipid homeostasis in a cohort of patients before and after antiviral therapy^23^. Among them, we identified a distinct subset of hepatokines modified by HCV clearance that could be implicated in liver-to-peripheral organ crosstalk. We thus postulated that infected hepatocytes could modulate the insulin sensitivity of non-infected tissues through the secretion of soluble factors. Here, we evaluated in another group of 23 lean, non-diabetic, CHC patients infected with different genotypes, the hepatic expression of some of these factors known to be expressed in the liver (ANGPTL4, ANGPTL6, IGFBP7, SEPP1, visfastin, chemerin and vaspin) (Table 1 and Fig. 1a). Among them, *ANGPTL4* mRNA expression positively correlated with HCV RNA and HOMA-IR (Fig. 1a–c). Such findings combined with our previous data showing that plasma ANGPTL4 levels are modified by DAA administration in HCV-infected patients^23^, suggest a possible role of ANGPTL4 in the pathogenesis of HCV-induced IR.

**Table 1:** Baseline characteristics of 23 chronic hepatitis C patients.

Males	18 (78%)
Age (years)*	37 (25–49)
BMI*	23.4 (19.0–24.5)
HCV RNA Log10 (IU/mL)*	6.3 (4.2–7.0)
HCV genotype	
1	14 (61%)
3	6 (26%)
4	2 (9%)
6	1 (4%)
Fasting plasma glucose (mmol/mL)*	4.9 (2.7–6.6)
Fasting plasma insulin (mIU/L)*	11.5 (2.1–23.6)
HOMA-IR*	2.8 (0.49–5.1)

*Data reported as median values (range).

### HCV-3a core protein expression in murine hepatocytes induces hepatic and muscle insulin resistance and increases *Angptl4* expression

**Figure 2:**
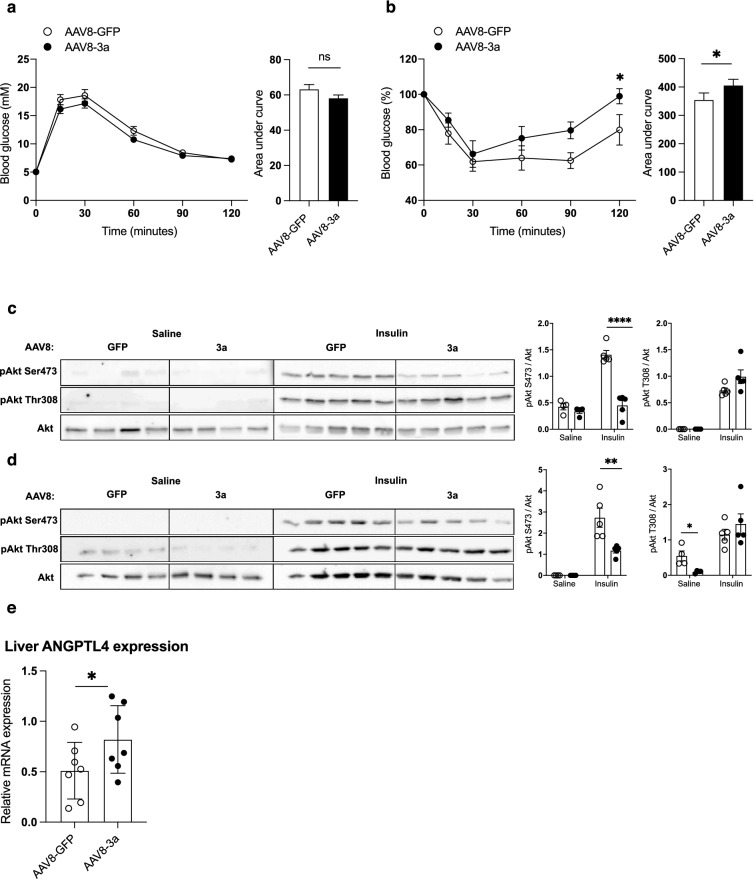
Overexpression of HCV-core protein in mice liver causes hepatic and muscle insulin resistance and increases ANGPTL4 expression. (**a**) Glucose tolerance test in mice infected with either AAV8-GFP or AAV8-3a for 3 months performed after overnight fasting; (**b**) Insulin tolerance test performed after 4 h of food deprivation; and (**c-d**) Representative immunoblots of pAkt Ser473, pAkt Thr308 and Akt total expression in the liver (**c**) and the gastrocnemius muscle (**d**) of AAV8-GFP and AAV8-3a mice challenged with insulin. (**e**) *Angptl4* mRNA expression in liver of mice expressing either the HCV-3a core or GFP in the hepatocytes. Quantification were performed on 4–5 mice/group. *p < 0.05, **p < 0.01 and ****p < 0.0001.

Previous studies have essentially provided mechanistic information regarding the hepatic contribution of HCV infection to IR. To address the role of HCV in peripheral IR, we developed an in vivo mouse model to recapitulate the liver-to-periphery crosstalk observed in HCV-infected patients. In order to avoid the production of viral particles and to totally rule out the possible direct effect of HCV particles on non-hepatocyte cells, we expressed exclusively in the hepatocytes a single HCV core protein rather than the full HCV genome. Indeed, a large number of studies have demonstrated that the expression of core protein of HCV genotype 3a (HCV-3a core) alone in hepatoma cells is able to reproduce the glucose alterations observed in HCV infection^12,28–31^. Mice were thus infected with the hepatotropic AAV8 encoding either HCV-3a core or green fluorescent protein (GFP, used as control) under the control of the albumin promoter (Supplementary Fig. S1a and b online). In this model, approximately 80% of the hepatocytes were infected by AAV8^32^, while the other screened tissues (heart, pancreas, adipose tissues and skeletal muscle) were not (Supplementary Fig. 1c and d online), supporting the hepatotropism of AAV8. Body weight, glycemia (Supplementary Fig. S1e and f), and glucose tolerance test (GTT) (Fig. 2a) were similar between AAV8-HCV-3a and AAV8-GFP animals. Fed plasma insulin levels tended to increase in AAV8-HCV-3a as compared to AAV8-GFP animals, albeit not significantly (Supplementary Fig. 1g online). However, AAV8-HCV-3a mice had marked IR as shown by insulin tolerance test (ITT) (Fig. 2b), a significant decrease of insulin-induced Akt-Ser473 phosphorylation in both liver and skeletal muscle and significant decrease of muscle Akt-Thr308 phosphorylation in basal conditions (Fig. 2c, d). Despite the fact that insulin-mediated Akt activation depends on two phosphorylated sites: Akt Ser473 and Akt Thr308, HCV-core only impaired Akt-Ser473 phosphorylation to induce IR. Recent studies have highlighted non-canonical pathways regulating Akt phosphorylation and downstream outcomes of these phosphorylations, suggesting that insulin-induced Akt phosphorylation can be more complex and thus reduced Akt-Ser473 phosphorylation may be sufficient to drive IR^33^. Overall, our observations show that the HCV core protein alone is able to induce IR not only in core-expressing but also in non-expressing tissues, such as skeletal muscle and that a potential endocrine effect of this viral protein may lead to peripheral IR. *Angptl4* mRNA levels were significantly increased in the livers of core-expressing mice as compared to control group (Fig. 2e). In addition, data from a compendium of single cell transcriptome in mouse liver showed that in the liver, *Angptl4* is almost exclusively expressed in the hepatocytes (Supplementary Fig. S2 online) (Tabula muris, https://tabula-muris.ds.czbiohub.org/), suggesting that the deregulation of expression induced by HCV might occur essentially in the hepatocytes. The lack of a reliable antibody against mouse ANGPTL4 and significant homology differences between mouse and human ANGPTL4 prevented us from using antibodies against human ANGPTL4, and thus fail to measure plasma ANGPTL4 levels in our mice.

### Expression of HCV-3a core protein in human hepatoma cells induces myotube insulin resistance partially through ANGPTL4

**Figure 3:**
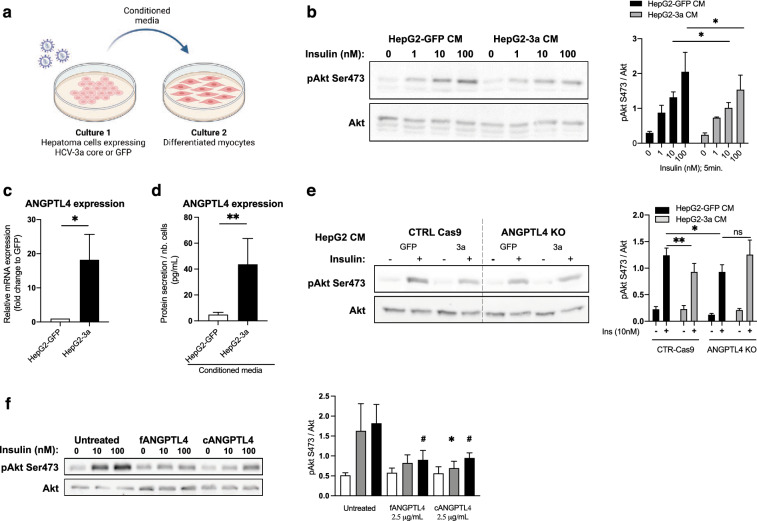
ANGPTL4 is a driver of muscle IR. (**a**) Differentiated C2C12 cells were treated for 24 h with CM of HepG2 cells transduced with either GFP or HCV‐3a core expressing lentivectors before being stimulated with insulin (1, 10 or 100 nM for 5 min) (created with BioRender.com). (**b**) Representative immunoblots and quantification of pAkt Ser473 and Akt total expression in C2C12 cells. (**c**) *ANGPTL4* mRNA expression and (**d**) ANGPTL4 secretory levels in HCV genotype 3a core-expressing HepG2 cells. (**e**) Representative immunoblots and quantification of pAkt Ser473 and total Akt expression of C2C12 cells treated with CM from non-edited (CTRL-Cas9) and edited (*ANGPTL4* KO) HepG2 cells transduced with GFP or HCV-3a core. *p < 0.05 and **p < 0.01. (**f**) Representative immunoblots and quantification of pAkt Ser473 and total Akt expression in human differentiated myocytes treated with different forms of human recombinant ANGPTL4 (2.5 µg/mL) for 24 h. Comparisons are represented as *insulin 10 nM and #insulin 100 nM. * or #* p* < 0.05.

In order to further assess the mechanisms whereby HCV induces peripheral IR, we used conditioned medium (CM) from HCV-core overexpressing hepatoma cells to treat differentiated myocytes in vitro (Fig. 3a). As observed in our in vivo mice model, myocytes treated with CM derived from HepG2 expressing HCV-3a core displayed a reduced Akt-Ser473 phosphorylation as compared to cells treated with control CM (Fig. 3b). Change of ANGPTL4 expression and secretion by HCV-3a core protein was assessed by measuring mRNA expression and protein content in the supernatant of hepatoma cells transduced with either HCV-3a core or GFP, respectively. Overexpression of HCV-3a core (as well as HCV-2a and 4 h but not 1b core, Supplementary Fig. S3a online) in HepG2 cells led to a significant increase of *ANGPTL4* mRNA levels and resulted in a tenfold increase (*p* = 0.003) of the secreted ANGPTL4 as compared to control (Fig. 3c, d). In addition, we confirmed a significant upregulation of *ANGPTL4* mRNA levels induced by the expression of HCV-3a core in a different hepatoma cell line—Huh-7 (Supplementary Fig. S3b online) and a positive effect (but significant only for 2a-JC1 chimera) of *ANGPTL4* mRNA levels promoted by the expression of HCV full-length genome (intra- and intergenotypic chimeras), possibly due to the reduced levels of HCV-core as compared to the lentiviral system previously documented in our laboratory^34^ (Supplementary Fig. S3c). We then evaluated the functional relevance of ANGPTL4 increased secretion in HCV-3a-mediated myotube IR. CRISPR-Cas9 technology was used to generate *ANGPTL4* KO cells (Supplementary Fig. S4 online). The expression of other members of the ANGPTL family (e.g. ANGPTL6 and 8, expressed in the liver) as well as several hepatokines known to be involved in glucose metabolism was not altered in *ANGPTL4* KO cells (Fig. S4e). As shown in Fig. 3e, CM of *ANGPTL4*-KO HepG2 cells transduced with HCV-3a core did not induce myotube IR. The direct role of ANGPTL4 in the pathogenesis of muscle IR was further confirmed by treating human differentiated myocytes with human ANGPTL4 recombinant protein. In circulation, ANGPTL4 can be found either in its native form, which corresponds to the full-length and unprocessed protein (flANGPTL4), or in its truncated forms, which result from the proteolytic cleavage of the protein into a N-terminal (nANGPTL4) and C-terminal (cANGPTL4) fragments^35,36^. The Luminex technology used to measure ANGPTL4 levels in the plasma of CHC patients and CM only allowed the detection of flANGPTL4 and cANGPTL4 fragments and not nANGPTL4 fragment. As our measurements indicated alterations of either flANGPTL4 or cANGPTL4, we focused on these two forms of the protein and evaluate their effects on muscle IR. We treated differentiated myocytes with 2.5 µg/mL of flANGPTL4 and cANGPTL4 for 24 h. Both forms induced an important decrease of insulin-induced Akt-Ser473 phosphorylation (Fig. 3f), suggesting that ANGPTL4 may directly alter insulin sensitivity in muscle cells.

### HCV-3a core protein induces upregulation of *ANGPTL4* through regulation of PPARγ

**Figure 4:**
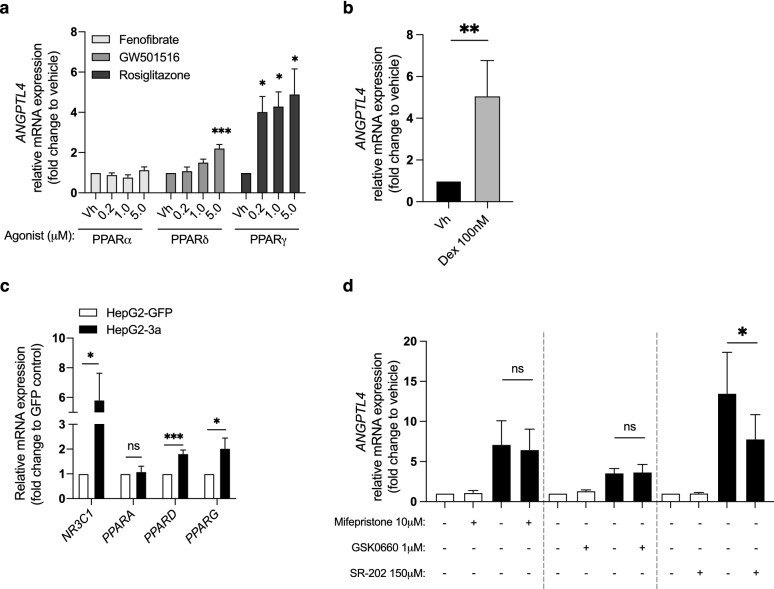
HCV induces upregulation of *ANGPTL4* expression via PPARγ. (**a, b**) *ANGPTL4* mRNA expression induced by PPAR (**a**) and GR agonists (**b**). (**c**) *NR3C1*, *PPARA*, *PPARD* and *PPARG* mRNA expression in levels in HCV genotype 3a core-expressing HepG2 cells. (**d**) Effect of GR antagonist (mifepristone) and PPAR antagonist (SR-202) in the core-induced expression of *ANGPTL4*. *p < 0.05, **p < 0.01 and ***p < 0.001.

To further understand the regulatory mechanisms involved in HCV-3a core-induced ANGPTL4 expression in hepatocytes, we focused our investigation on transcription factors known to contribute to the regulation of *ANGPTL4* expression. Several studies have demonstrated that glucocorticoid receptor (GR, coded by *NR3C1* gene) and PPARs are transcriptional regulators of *ANGPTL4* expression in several cell types^37–44^. Thus, as a first control experiment, we aimed at validate these effects in our system by treating HepG2 with PPAR agonists and glucocorticoids. Treatment with GR, PPARδ and PPARγ agonists but not with PPARα agonist resulted in a significant upregulation of *ANGPTL4* mRNA levels in HepG2 (Fig. 4a, b). These findings encouraged us to further investigate the regulation of *ANGPTL4* in this cell model. In addition, expression of HCV-3a core led to a significant upregulation of *NR3C1, PPARD* and *PPARG* expression (Fig. 4c) and confirmed the previously published PPAR increased expression^45^. To determine whether the activation of one or several of these transcription factors could be involved in the HCV-driven upregulation of *ANGPTL4*, we treated HCV-3a core-expressing HepG2 cells with selective antagonists for each transcription factor and measured the *ANGPTL4* mRNA level. While the inhibition of GR activity with mifepristone and PPARδ with GSK0660 did not modify the core-induced expression of *ANGPTL4*, treatment with a PPARγ antagonist (SR-202) led to a significant downregulation of *ANGPTL4* mRNA levels in HCV-3a expressing HepG2, suggesting that this transcription factor might be involved in HCV-driven *ANGPTL4* upregulation (Fig. 4d).

## Discussion

The fact that (1) HCV primarily infects and replicates in hepatocytes and (2) HCV affects insulin sensitivity in peripheral organs has led to the hypothesis that infected cells would release factors able to induce IR in uninfected extrahepatic tissues. The identification of these soluble factors should not only improve our knowledge of the role of the liver in inter-organ communication, but also provide new therapeutic options to treat IR and diabetes in HCV infection and in other etiologies as well, such as metabolic syndrome and obesity.

Over the years, several soluble factors involved in inter-organ communication have been implicated in the pathogenesis of T2D^46,47^. We previously identified a distinct subset of metabolic active hepatokines in the plasma of CHC patients, potentially involved in liver-to-periphery crosstalk and thus peripheral IR^23^. Among them, we found here that *ANGPTL4* mRNA expression in the liver positively correlates with HCV RNA and IR degree, suggesting that ANGPTL4 might be one of the mediators of HCV-driven peripheral IR. This possibility was then further reinforced by publicly available data from a single cell transcriptome in mouse liver reporting that ANGPTL4 in the liver is almost exclusively expressed in the hepatocytes (cells in the liver that are infected by HCV), and by the observation that expression of HCV-3a core in hepatoma cells as well as in mouse liver promotes mRNA expression and secretion of ANGPTL4. In addition, we also demonstrated that *ANGPTL4* mRNA overexpression can be induced by different HCV genotypes. The observation that the HCV-1b core had no effect on *ANGPL4* expression suggests that, although IR occurs in all genotypes^21,48^, the underlying mechanisms may be genotype specific, as previously shown^12^. PPARs and GR are the main recognized transcription factors involved in the regulation of *ANGPTL4*^35,44,49,50^. Our results indicate that HCV-3a core-induced ANGPTL4 expression in HepG2 is, at least partly, regulated by PPARγ, while GR and PPARδ do not seem to be involved.

ANGPTL4 is a secreted glycoprotein that belongs to a superfamily of ANGPTLs, and has been implicated in a variety of diseases and biological processes, including cardiovascular disease^51,52^, obesity^53^, diabetes^54,55^, angiogenesis and hypoxia^56,57^. ANGPTL4 is highly expressed in metabolic tissues, such as adipose tissue and liver and plays a role on the regulation of plasma triglycerides (TG) clearance, through inhibition of lipoprotein lipase, a rate-limiting enzyme responsible for the hydrolysis of TG into free fatty acids^58–60^. Genetic studies in humans have revealed a positive association between ANGPTL4 and glucose intolerance. Carriers of E40K mutation (ANGPTL4 inactive variant) in addition to lower plasma TG, have also less risk of developing T2D^54,61–63^, and are more protected against metabolic alterations associated with obesity than noncarriers^64^. Accordingly, other studies demonstrated that *ANGPTL4* transcriptional levels in visceral adipose tissues and liver, as well as circulating levels are positively associated with impaired glucose metabolism in obese subjects^55,65,66^. Genetic ablation of *ANGPTL4* in the adipose tissue and liver of rodents has further confirmed the role of ANGPTL4 on lipid and glucose metabolism^67–69^. For instance, ANGPTL4 loss-of-function in murine hepatocytes resulted in reduced circulating TG levels, increased glucose tolerance and insulin sensitivity characterized by enhanced hepatic and muscle Akt phosphorylation^69^. This observation is in agreement with our results showing that recombinant ANGPTL4 impairs Akt activation in muscle cells, and that ANGPTL4 ablation in hepatoma cells protects muscle cells from CM-3a core induced IR. ANGPTL4 can be found in circulation in three distinct forms: (1) native and unprocessed protein (flANGPTL4), (2) N-terminal (nANGPTL4) fragment, and (3) C-terminal (cANGPTL4) fragment; these last two resulting from the proteolytic cleavage of the native protein^35,36^. These protein forms are associated with different biological functions: flANGPTL4 and nANGPTL4 act in the inhibition of lipoprotein lipase activity, while flANGPTL4 and cANGPTL4 stimulate adipose tissue lipolysis, promote energy expenditure, and have been associated with antiangiogenic functions^70–74^. As far as we know, no study has ever provided mechanistic insights towards the role of the specific forms of ANGPTL4 on glucose homeostasis. Our results demonstrated an increased hepatic flANGPTL4 and/or cANGPTL4 secretion induced by HCV-core expression, and that treatment of differentiated myocytes with both flANGPTL4 and cANGPTL4 recombinant proteins results in Akt inactivation, suggesting a possible direct action of HCV-mediated ANGPTL4 secretion in the regulation of muscle insulin sensitivity. Based on our results muscle IR seems to be induced by the presence of higher levels of cANGPTL4 either displayed in the native (flANGPTL4) or cleaved (cANGTPL4) form. Similarly to our in vitro and mouse HCV-3a core models, cANGPTL4-induced muscle IR is driven by Akt Ser437 phosphorylation but no alterations were observed in Akt Thr307 phosphorylation. The detailed mechanisms by which cANGPTL4 decreases Akt phosphorylation need to be further investigated. A couple of studies have demonstrated that cANGPTL4 interacts with integrins and stimulates integrin-mediated signaling interfering with PI3K cascade^75–77^. In particular, high fat diet-induced muscle IR has been associated with α2β1 integrin activation, suggesting a role of integrins in the regulation of insulin sensitivity^78^.

In the context of CHC, we show here for the first time a link between muscle IR and HCV-core induced ANGPTL4 expression and secretion. In addition to HCV-mediated IR, a recent study has also associated higher ANGPTL4 serum levels with advanced fibrosis, and disease progression to cirrhosis and HCC^79^. We previously observed increased ANGPTL4 serum levels in CHC patients without significant fibrosis before antiviral treatment^23^, indicating that ANGPTL4 expression levels are upregulated since early stages of chronic liver disease.

Overall, our work shows that HCV induces peripheral IR, probably via the release of soluble factors by infected cells. Uncovering the mechanisms whereby HCV induces IR may lead to an improved management of hepatogenous alterations of glucose homeostasis, not only in the setting of HCV infection but also in other liver disorders characterized by IR, such as non-alcoholic fatty liver disease. Our investigations identified ANGPTL4 as an HCV-induced hepatokine that plays a key role in the IR of muscle cells. A better understanding of its function in the pathogenesis of HCV-mediated glucose abnormalities may allow the development of new treatment or biomarker in the diagnosis and prognosis of glucose metabolic disorders.

## Methods

### Primers, antibodies and plasmids

Primers, antibodies and plasmids are described in Supplementary Materials.

### Cell culture

Human hepatoma cell lines (HepG2 and Huh-7) and HEK293T cells were cultured in low glucose Dulbecco’s modified Eagle’s medium (DMEM), supplemented with 10% fetal calf serum (FCS), glutamine (2 mM), penicillin–streptomycin (100 U/mL) (all from Gibco). C2C12 cells (murine myoblasts) were grown in DMEM-Glutamax (4.5 g/L glucose), supplemented with 10% FCS, and penicillin–streptomycin (100 U/mL), and differentiated in DMEM-Glutamax (4.5 g/L glucose), supplemented with 2% FCS, and penicillin–streptomycin (100 U/mL) for 7 days. Human primary satellite cells were grown until confluency in proliferation medium by supplementing Ham's F-10 medium with 2% FCS and 2% serum substitute (Ultraser G, PALL life sciences), glutamine (2 mM), penicillin–streptomycin (100 U/mL), and differentiated in DMEM supplemented with 2% FCS, glutamine (2 mM), penicillin–streptomycin (100 U/mL). Human differentiated myocytes were used for experiments after 5 to 7 days of differentiation. All donors were lean and were not diagnosed for T2D, neoplasia, chronic inflammatory diseases, or HIV, HBV and HCV infection. Biopsies were taken from *vastus lateralis* or from *rectus abdominis* muscles during planned surgeries (Diomede experimental protocol). All procedures were approved by the French Ethical Committee SUD EST IV (Agreement #12/111 A 13–02) and performed according to the French legislation (Huriet’s law). Muscle differentiation was characterized by the fusion of myoblasts into polynucleated myotubes visualized microscopically^80^.

### ANGPTL4 knockout HepG2 cell generation

For the generation of *ANGPTL4* KO HepG2 cells, *ANGPTL4* targeting guide RNA were designed using the Synthego CRISPR Design Tool (https://www.synthego.com/products/bioinformatics/crispr-design-tool). The guide RNA 5′-CACCCCCTCCCCAGACACAACTCA-3′ targeting the exon 2 of *ANGPTL4* was cloned in the plasmid LentiCRISPRv2 containing the Cas9 cassette. Lentiviral particles coding for the guide RNA were produced as described below. After transduction, HepG2 cells were treated with 4 mg/ml puromycin to select for cells with LentiCRISPRv2-gRNA ANGPTL4 or LentiCRISPRv2 (used as control) for 5 days before cell clone selection.

Gene editing was evaluated using GeneArt^®^ Genomic Cleavage Detection Kit (Thermo Fisher Scientific, USA), according to the manufacturer instructions. This technique allows the detection of locus specific cleavage of genomic DNA. A 551 bp DNA fragment encompassing the site targeted by the gRNA was amplified by PCR (see Supplementary Materials for primer sequences). The PCR product was then denatured at 90 °C for 5 min and re-annealed to generate mismatches. During re-annealing, strands with genomic insertions or deletions (indel) caused by CRISPR-Cas9 can anneal to either unmodified strands or with a different indel. Mismatches were then cleaved by a mismatch-specific endonuclease. The resulting bands were analyzed by agarose gel electrophoresis. To further confirm the indel, PCR amplicon was Sanger sequenced (Fasteris).

### Lentiviral production

Lentiviral particles were produced by transient transfection in HEK293T cells, collected in culture supernatant 48 h after calcium phosphate transfection, filtered through 0.45 µm pore-sized polyvinylidene difluoride membrane, and concentrated by ultracentrifugation at 20’000 rpm for 90 min. Viral titers were estimated by real-time PCR^81^. HepG2 or Huh-7 cells were transduced with 1 MOI of lentiviral vectors and RNA was extracted for further studies.

### Myocytes treatment with conditioned media derived from hepatoma cells expressing the HCV-core protein

HepG2 were transduced with lentiviral vectors encoding either HCV-3a core protein or GFP (control). Conditioned media (CM) was prepared by changing the media to FCS-depleted DMEM supplemented with 0.5% bovine serum albumin (free fatty acid (FFA) depleted) 48 h after transduction. After 24 h, CM was harvested and filtered through 0.22 µm pore-sized polyvinylidene fluoride membrane. Differentiated myocytes were treated with HepG2 CM for 24 h, and then subjected to insulin stimulation for 5 min at 37 °C.

### Treatment of human skeletal muscle cells with recombinant ANGPTL4

Human differentiated myocytes were treated with 2.5 µg/mL of recombinant ANGPTL4 (full-length, and c-terminal, R&D) for 24 h. After treatment media was replaced, myocytes were stimulated with insulin for 5 min and cell lysates were harvested to evaluate Akt phosphorylation.

### Human studies

RNA samples obtained from liver biopsies were used for RT-qPCR analysis. Thirteen samples were obtained from patients that were enrolled in a multicenter trial conducted by the DITTO-HCV (Dynamically Individualized Treatment of Hepatitis C Infection and Correlates of Viral/Host Dynamics) study group^82^, ten were recruited at the University Hospital of Geneva. Written informed consent was obtained from each participating patient. Ethical committees at each study centre approved the treatment study (Number 02-070). For all patients, histological and biochemical data (fasting plasma glucose and insulin) were collected. HOMA-IR was calculated as fasting glucose in mmol/L x fasting insulin in mIU/mL)/22.5. BMI was calculated from weight and height (BMI = weight (kg)/height^2^ (m^2^)). Patients with BMI > 25 kg/m^2^ or diabetes were excluded from the analysis. Total RNA from liver samples was extracted using the AllPrep DNA/RNA Mini Kit (Qiagen). The quality of the RNA was assessed using an Agilent 2100 Bioanalyser (Agilent Technologies).

### Animals

Two months C57BL/6J males were infected with Adeno-Associated Virus serotype 8 (AAV8) (retro-orbital injection) carrying either the HCV-3a core or GFP genes under the control of albumin promoter (pAAV-ALB-core3a-IRES-GFP and pAAV-ALB-GFP)^32^. To mimic a chronic infection, the animals received two injections of AAV8, a first injection at 2-month-old (5 × 10^11^gc/mL) and a second injection 2 months later (1 × 10^11^gc/mL). Metabolic tests were performed 3 months after the first AAV8 injection (see Supplementary Fig. S1b online). Animals were sacrificed using isoflurane anaesthesia followed by rapid decapitation. Blood and tissues were collected and stored at − 80 °C. All experiments were conducted in accordance with the Swiss guidelines for animal experimentation and following the ARRIVE guidelines, and were ethically approved by the Geneva Health head office (Authorization No. GE-143-17).

### Metabolic tests

Glucose (1.5 g/kg) was administered intraperitoneally, after overnight starvation; and insulin (0.75U/kg), after 4 h of food deprivation. Glycemia was measured from tail blood every 30 min during 2 h using glucose testing strips (Accu-chek, Aviva). To investigate insulin signaling in organs, mice were injected intraperitoneally 20 min before sacrifice with 5U/kg of insulin (or 0.9% sodium chloride), after 3 h of food deprivation. Blood insulin levels were measured using Ultrasensitive Mouse Insulin ELISA (Mercodia).

### Immunohistochemical analysis

Fresh tissues were fixed in 4% paraformaldehyde, embedded in paraffin, cut 3 μm thick and mounted on glass slides (Superfrost Plus, Thermo Fisher Scientific). Antigen retrieval was performed with a Pascal high-pressure chamber in 10 mM citrate buffer, pH 6.0. Sections were blocked with hydrogene peroxide for 5 min, incubated with rabbit anti-GFP antibody for 30 min at room temperature, and subsequently incubated with anti-rabbit-horseradish peroxidase (HRP) and 3,3′-diaminobenzidine for revelation.

### Immunoblot analysis

Tissues and cells were lysed in ice-cold RIPA buffer (NaCl 300 mM, NP40 2%, SDS 0.20%, deoxycholic acid 1%, Tris HCl 100 mM pH 7.4) supplemented with protease (phenylmethylsulfonyl fluoride and protease inhibitor cocktail, Roche) and phosphatase (sodium fluoride, sodium orthovanadate and sodium pyrophosphate decahydrate) inhibitors. Protein content was determined by Pierce BCA protein assay (Thermo Scientific). Equal amounts of proteins were separated by 10% SDS-PAGE and transferred onto nitrocellulose membranes. Proteins were detected with specific primary antibodies and HRP conjugated secondary antibodies (see Supplementary Materials) using enhanced chemiluminescence reagent. Quantifications were performed using the ImageJ software.

### RNA isolation, reverse transcription and real-time PCR

Total intracellular RNA was extracted using the Nucleospin RNA II Kit (Macherey–Nagel AG). cDNA was synthesized from 500 ng total RNA with Transcriptor Universal cDNA master (Roche Diagnosis). The primers used are described in Supplementary Materials. Relative quantification of mRNA transcripts was performed by qRT-PCR as described^83^, using either *EEF1A1* or cyclophilin as housekeeping genes.

### ANGPTL4 measurements

Human ANGPTL4 levels were measured using a bead-based multiplex (Human magnetic luminex assay, R&D systems). Samples and standards were run in duplicate.

### Statistical analysis

Statistical analysis was performed using Graphpad Prism 7 software. Results are expressed as means ± SEM of at least 3 independent experiments or at least 4 different animals per group. Results were analyzed by Student’s t-test or two-way ANOVA test when more than 2 groups or multiple time points were analyzed. Correlation analyses were performed by using the Spearmen’s correlation coefficient test. A value of *p* < 0.05 was considered significant. *p < 0.05; **p < 0.01; ***p < 0.001.

## Supplementary Information

Supplementary Information.

## Data Availability

All data generated or analyzed during this study are available from the corresponding author on reasonable request.
