# Supplementary material for: ANGPTL4 is a potential driver of HCV-induced peripheral insulin resistance
Source: Sci Rep. 2023 Apr 25;13:6767. doi: 10.1038/s41598-023-33728-5 (PMC10130097; doi:10.1038/s41598-023-33728-5)
Supplement: Supplementary file 1 — Supplementary Information. [file 41598_2023_33728_MOESM1_ESM.docx]

**Supplementary Materials**

**ANGPTL4 is a potential driver of HCV-induced peripheral insulin resistance**

Diana Gomes, Cyril Sobolewski, Stéphanie Conzelmann, Tifany Schaer, Etienne Lefai, Dulce Alfaiate, Eirini Tseligka, Nicolas Goossens, Caroline Tapparel, Francesco Negro, Michelangelo Foti and Sophie Clément

Table of Contents

[I- Supplementary materials and methods 2](#_Toc130306899)

[Antibodies, primers, plasmids and reagent 2](#_Toc130306900)

[Primary and secondary antibodies 2](#_Toc130306901)

[Human and mouse PCR primers 2](#_Toc130306902)

[Plasmids and constructs 3](#_Toc130306903)

[Specific reagents 3](#_Toc130306904)

[II- Supplementary Figure 4](#_Toc130306905)

[Supplementary Figure S1 4](#_Toc130306906)

[Supplementary Figure S2 5](#_Toc130306907)

[Supplementary Figure S3 6](#_Toc130306908)

[Supplementary Figure S4 7](#_Toc130306909)

[Supplementary Figure S5 8](#_Toc130306910)

[Supplementary Figure S5 Cont. 9](#_Toc130306911)

[Supplementary Figure S6 11](#_Toc130306912)

[Supplementary Figure S6 Cont. 12](#_Toc130306913)

[Supplementary Figure S6 Cont. 13](#_Toc130306914)

[Supplementary Figure S7 14](#_Toc130306915)

[Supplementary Figure S8 15](#_Toc130306916)

[Supplementary Figure S9 16](#_Toc130306917)

[III- Supplementary references 17](#_Toc130306918)

I- Supplementary materials and methods

Antibodies, primers, plasmids and reagent

Primary and secondary antibodies

| **Targeted protein** | **Host** | **Clone** | **Provider** |
| --- | --- | --- | --- |
| pAkt Ser473 | Rabbit | 193H12 | Cell signaling |
| pAkt Thr308 | Rabbit | C31E5E | Cell signaling |
| Akt2 | Rabbit | 5B5 | Cell signaling |
| HCV-core | Mouse | C7-50 | Covalab (Lyon France) |
| β-cytoplasmic actin | Mouse | C4 | A gift from C. Chaponnier, Geneva, Switzerland |
| GFP | Rabbit | D5.1 | Cell signaling |
| HRP-conjugated anti-mouse | Goat |  | Bio-Rad Laboratories AG (Cressier, Switzerland) |
| HRP-conjugated anti-rabbit | Goat |  | Bio-Rad Laboratories AG (Cressier, Switzerland) |

Human and mouse PCR primers

| **Primers** | **Forward** | **Reverse** |
| --- | --- | --- |
| Human ANGPTL4 | agcctgcagacacaactcaa | ctaggtgcttgtggtccagg |
| Human ANGPTL6 | GATGGCTTCTCCCTGGAACC | ACAGGGCACAGTTACCAGAA |
| Human ANGPTL8 | GGCAAGVVTGTTGGAGAC | TGTCCCGTAGCACCTTCTGT |
| Human Vaspin | GCCAGGTGGAAACATGAGTT | CCCTCATCAGGAAGGATGAA |
| Human IGFBP7 | ATCCCGACACCTGTCCTCAT | CCCAGCCAGTTACTTCATGCT |
| Human Chemerin | AACTGGGCTCTGAGGACAAA | CTTGGAGAAGGCGAACTGTC |
| Human Visfatin | ggccacaaattctAGAGAGCAG | ccaagtGagcagaTgctcctat |
| Human SEPP1 | GCACCRRGGCAGCAGTGAGCTT | ATGAAGGCCTGGAGGAGCAGGA |
| Human FGF-21 | ATGGATCGCTCCACTTTGACC | GGGCTTCGGACTGGTAAACAT |
| Human LECT2 | gagtgtgggggcaactctaa | tccggatctcattggaagac |
| Human EEF1A1 | AGCAAAAATGACCCACCAATG | GGCCTGGATGGTTCAGGATA |
| Mouse cyclophilin | CAAATGCTGGACCAAACACAA | GCCATCCAGCCATTCAGTCT |
| HCV-core | TCCTAAACCTCAAAGAAAAACCAAA | TCCTGTGGGCGGCG |
| Mouse ANGPTL4 | CAAAACAGCAAGATCCAGCA | CCTCTTTCCCCTCGAAGTCT |
| Primers for GeneArt® Genomic Cleavage Detection Kit | GCCTCAGCGGATGGAGATTT | AGGTGGACCTGACAAGGAGA |

Plasmids and constructs

| **Plasmid name** | **Plasmid insert** | **Backbone** | **References/sources** |
| --- | --- | --- | --- |
| pLenti-GFP / pLenti-HCV-3a core | GFP / HCV-2a core / HCV-3a core | 2K7 (Invitrogen AG, Basel Switzerland) | (1) |
| LentiCRISPRv2 | Cas9 |  | A gift from Feng Zhang, Addgene plasmid # 52961 (2) |
| pFK-J6/C3 (Jc1) | Full length HCV genotype 2a |  | A gift from R. Bartenschlager, Heidelberg, Germany (3) |
| pFK-H77/C3 | Full length HCV genotype 1a |  | A gift from R. Bartenschlager, Heidelberg, Germany (3) |
| pFK-JFH1/Con1 | Full length HCV genotype 1b |  | A gift from R. Bartenschlager, Heidelberg, Germany (3) |
| pFK-452/C6 | Full length HCV genotype 3a |  | A gift from R. Bartenschlager, Heidelberg, Germany (3) |
| pED43/JFH1γ(A2819G/A3269T) | Full length HCV genotype 4a |  | A gift from J. Bukh, Copenhagen, Denmark (4) |

Specific reagents

| **Reagents** | Company | Reference |
| --- | --- | --- |
| GSK 0660 (PPARδ antagonist) | Tocris Bioscience | 1014691-61-2 |
| SR 202 (PPARγ antagonist) | Tocris Bioscience | 76541-72-5 |
| Rosiglitazone (PPARγ agonist) | Tocris Bioscience | 122320-73-4 |
| Fenofibrate (PPARα agonist) | Tocris Bioscience | 49562-28-9 |
| GW 501516 (PPARδ agonist) | Tocris Bioscience | 317318-70-0 |
| Mifepristone (glucocorticoid receptor antagonist) | Tocris Bioscience | 84371-65-3 |
| Dexamethasone | Sigma | D2915 |
| Recombinant ANGPTL4 (full-length) | R&D systems | AF3485 |
| Recombinant ANGPTL4 (c-terminal) | R&D systems | 3485-AN |
| Insulin Humalog | Lilly | VL7510 |
| Ultrasensitive Mouse Insulin ELISA | Mercodia | 10-1249-01 |
| Customized human magnetic luminex assay | R&D systems |  |

II- Supplementary Figure

Supplementary Figure S1

**
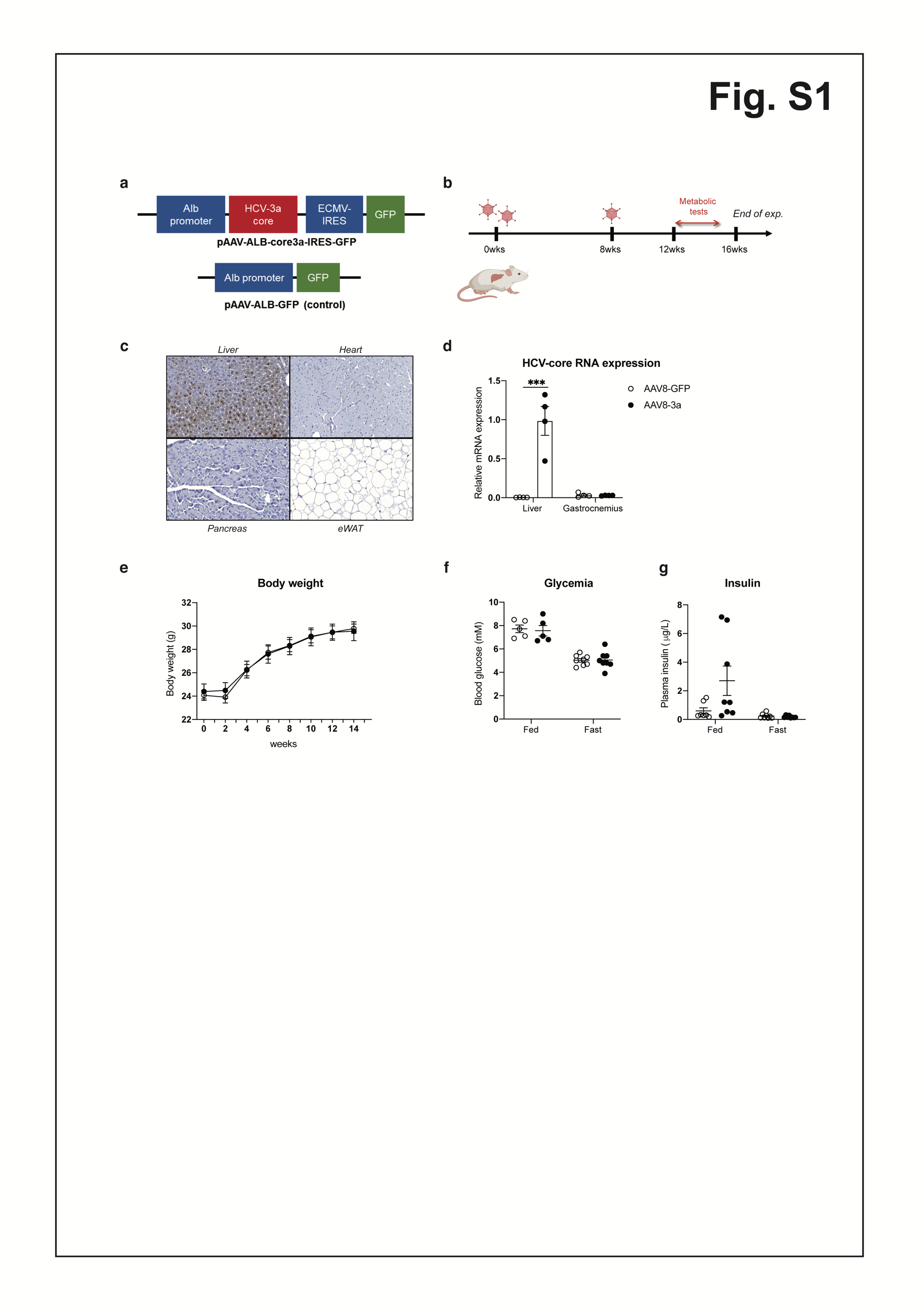
**

**HCV-mouse model characterization.** (**a**) Adeno-associated virus serotype 8 (AAV8) vectors used in the study; (**b**) experimental design of mice injection (created with BioRender.com); (**c**) GFP immunohistochemical staining of liver, heart, pancreas and epididymal white adipose tissue (eWAT) of AAV8-3a infected mouse; (**d**) HCV-core RNA expression in liver and gastrocnemius of mice; (**e**) body weight and (**f**) blood glycemia and (**g**) plasma insulin in fed and fasting conditions.

Supplementary Figure S2

**
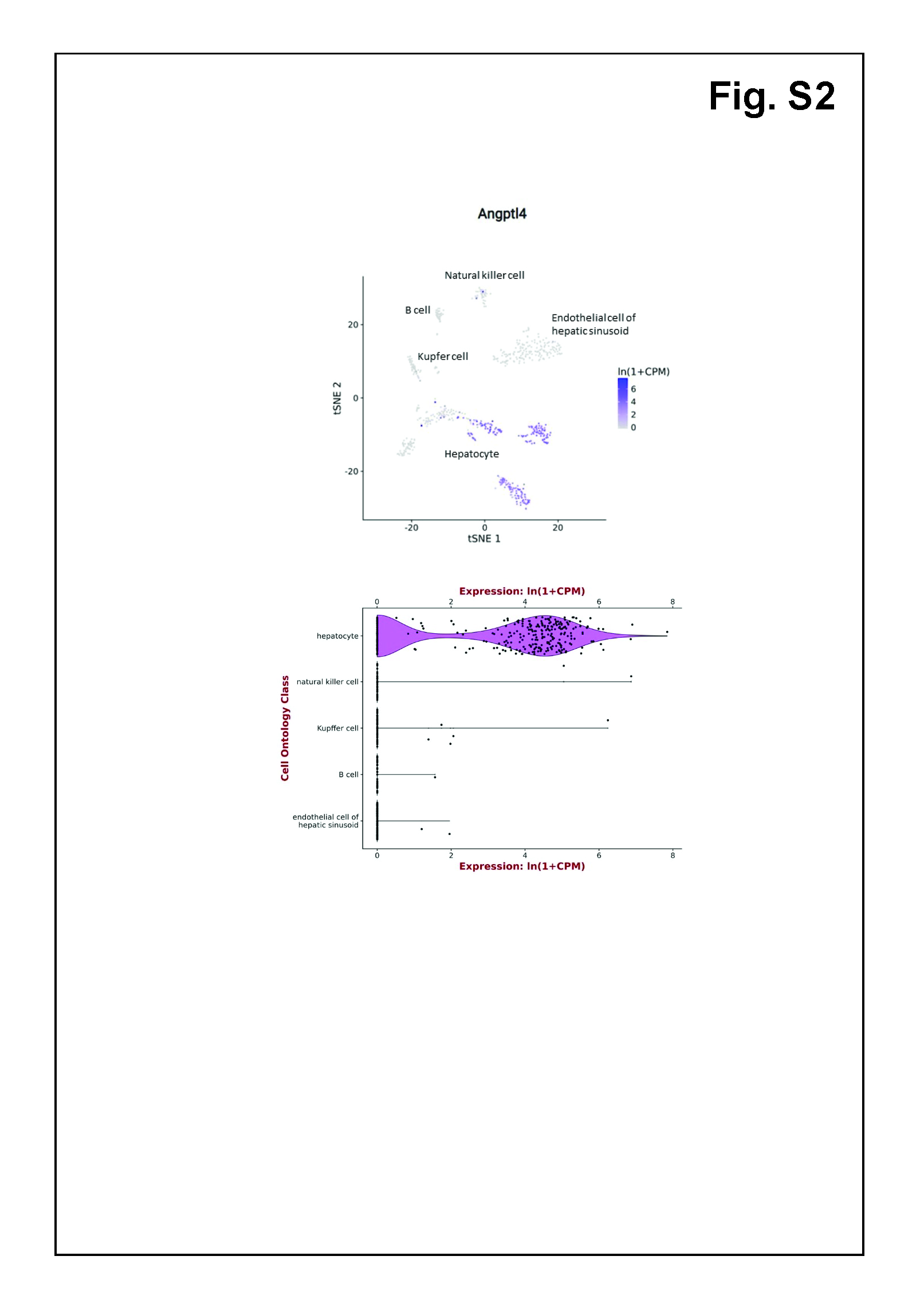
**

***ANGPTL4* gene expression in each cell type of the liver.** Publicly available data at Tabula muris, <https://tabula-muris.ds.czbiohub.org/>. t-SNE map and cell ontology class representation highlighting the expression of *ANGPTL4* in the hepatocytes.

Supplementary Figure S3


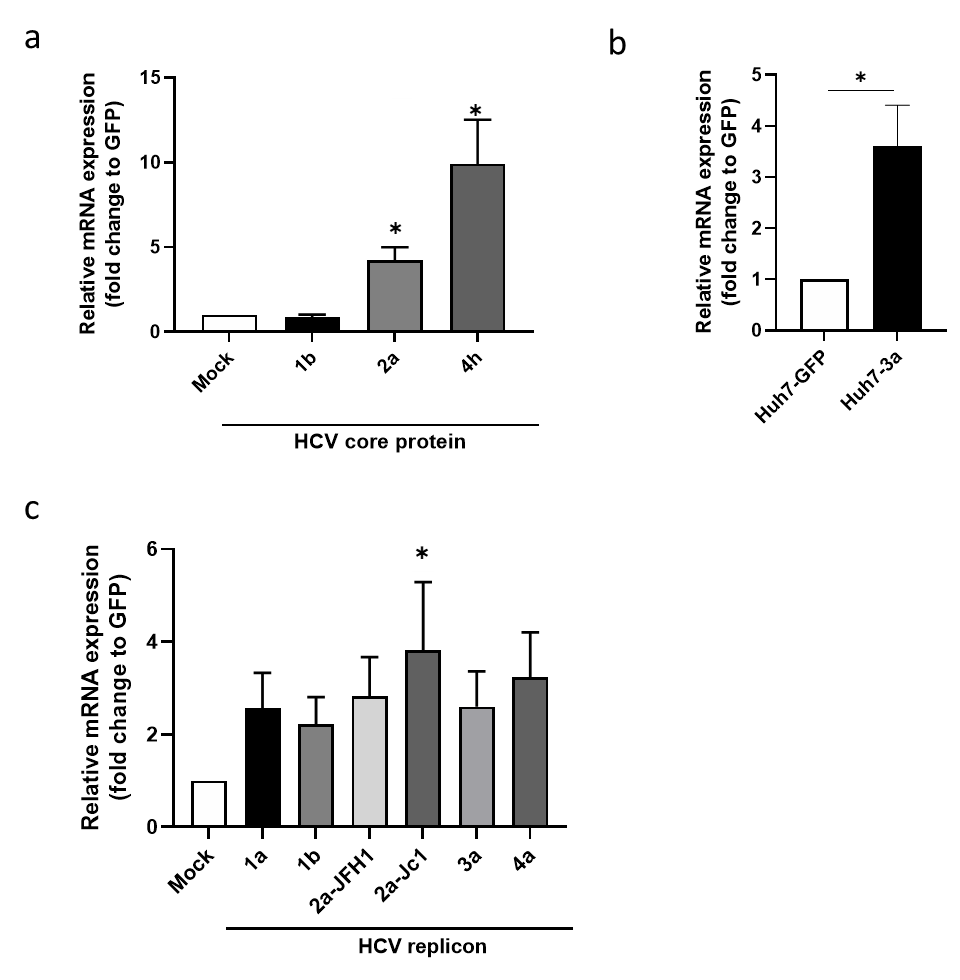


*ANGPTL4* mRNA expression in (**a**) HepG2 cells transduced with either GFP, HCV‐1b core, HCV‐2a core and HCV‐4h core; (b) Huh-7 cells transduced with either GFP or HCV‐3a core; **(c)** Huh-7 cells transfected with different HCV intra- and intergenotypic chimeras.

Supplementary Figure S4


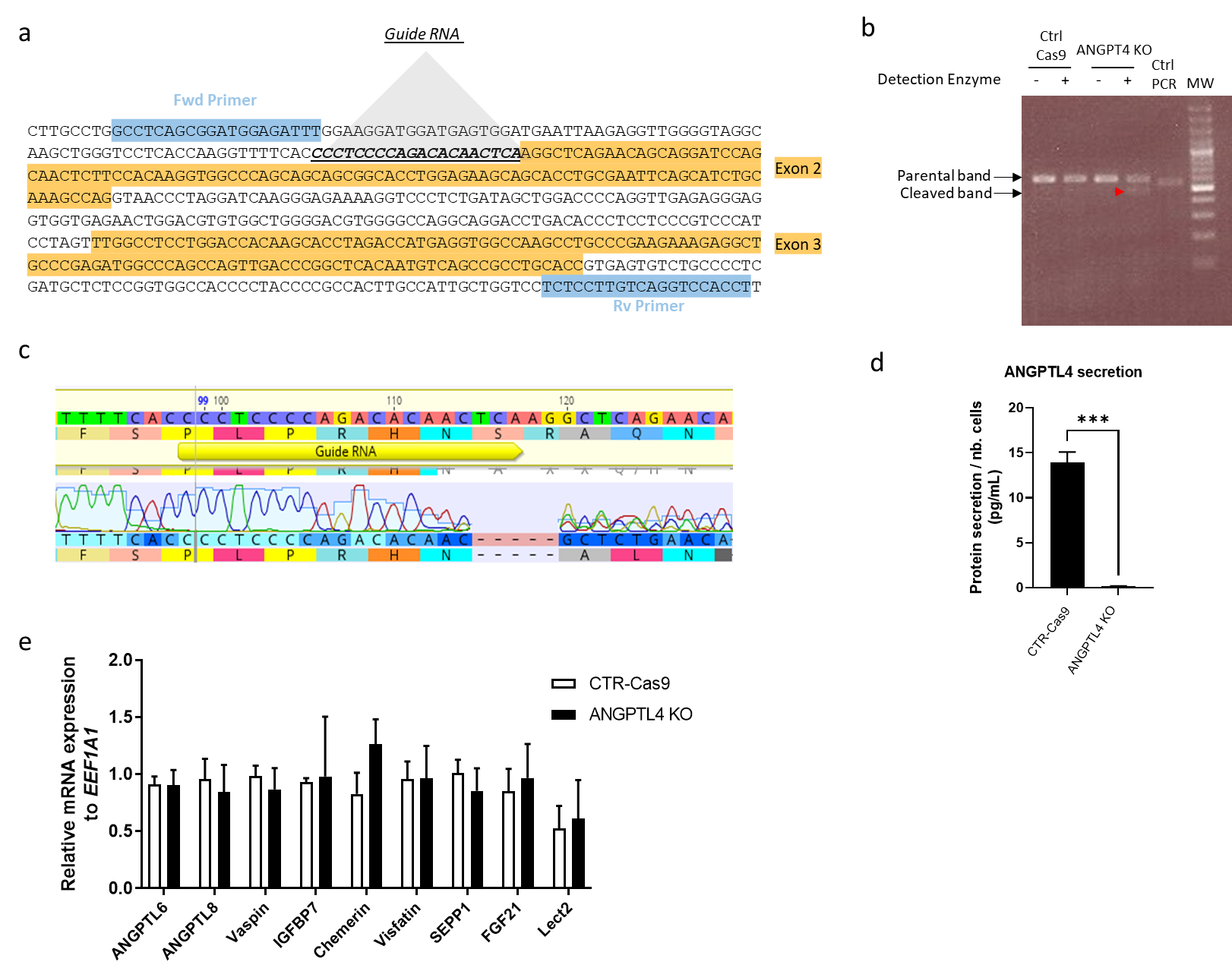


**Characterization of ANGPTL4 KO HepG2 cell lines.** (**a**) Sequence of the PCR amplified region encompassing the guide RNA target in exon 2 of human *ANGPTL4* gene. The positions of the two primers (Fwd and Rv primers) used for PCR amplification are highlighted in blue. Exons 2 and 3 are shown in yellow. (**b**) Representative agarose gel showing that PCR products of the polyclonal pool of cells transduced with ANGPTL4 guide RNA display a cleavage band (red arrows), whereas DNA from Cas9 expressing cells (control) is not cleaved. (**c**) Sanger PCR chromatogram of one clone of KO cells (clone 2-3) showing a 6bp deletion when compared with the wildtype sequence. The corresponding amino acid sequence is shown below the nucleotide codon sequences. (**d**) ANGPTL4 secretory levels in non-edited (CTRL-Cas9) and edited (ANGPTL4 KO) HepG2 cells. **(e)** mRNA expression of some hepatokines in non-edited (CTRL-Cas9) and edited (ANGPTL4 KO) HepG2 cells.

Supplementary Figure S5

**a**


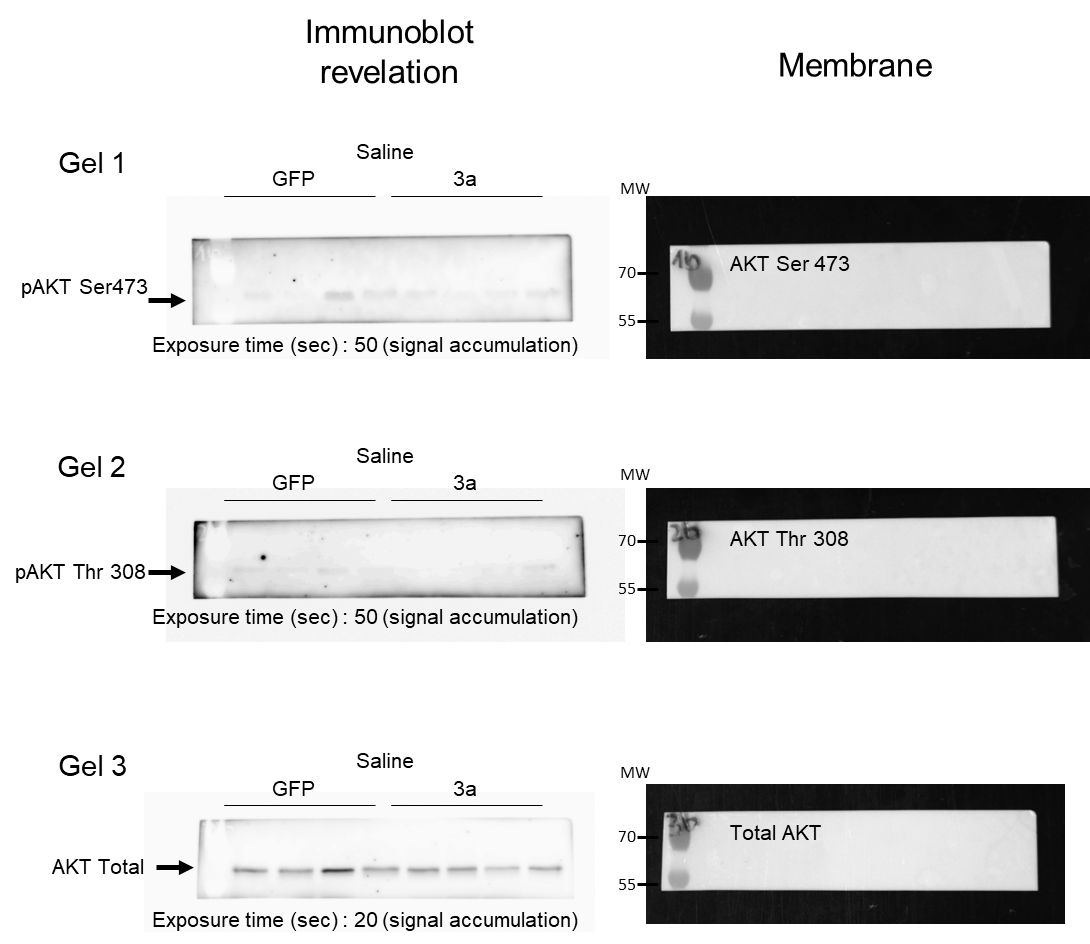


Supplementary Figure S5 Cont.

**b**


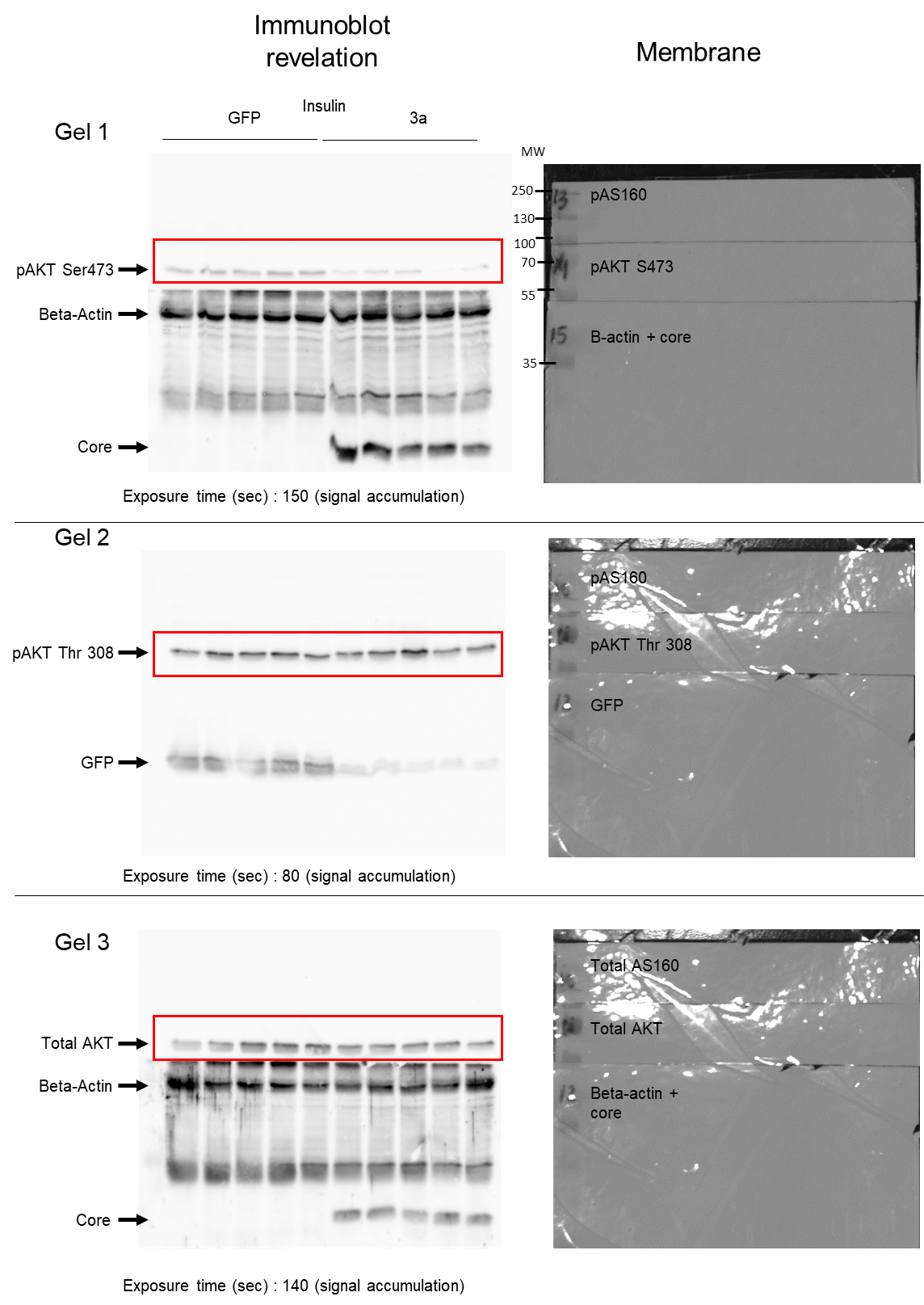


**Full immunoblots related to Figure 2c. (a)** Liver samples of mice treated with saline (0.9% NaCl) were loaded on 3 separate SDS-PAGE gels for Akt signaling analysis. Membranes were cut prior blotting with the different antibodies: total Akt, pAkt (Ser473 or Thr307). **(b)** Liver samples of mice challenged with insulin were loaded on 3 separate SDS-PAGE gels for Akt signaling analysis**.** Membranes were cut prior blotting with the different antibodies: total Akt, pAkt (Ser473 or Thr307), AS160, HCV-core, GFP and beta-actin. Beta-actin was used as loading control, and HCV-core and GFP antibodies were used to determine HCV-core and GFP expression levels induced by AAV8. Only the bands highlighted by a red rectangle are shown in the final figure.

Supplementary Figure S6

**a**


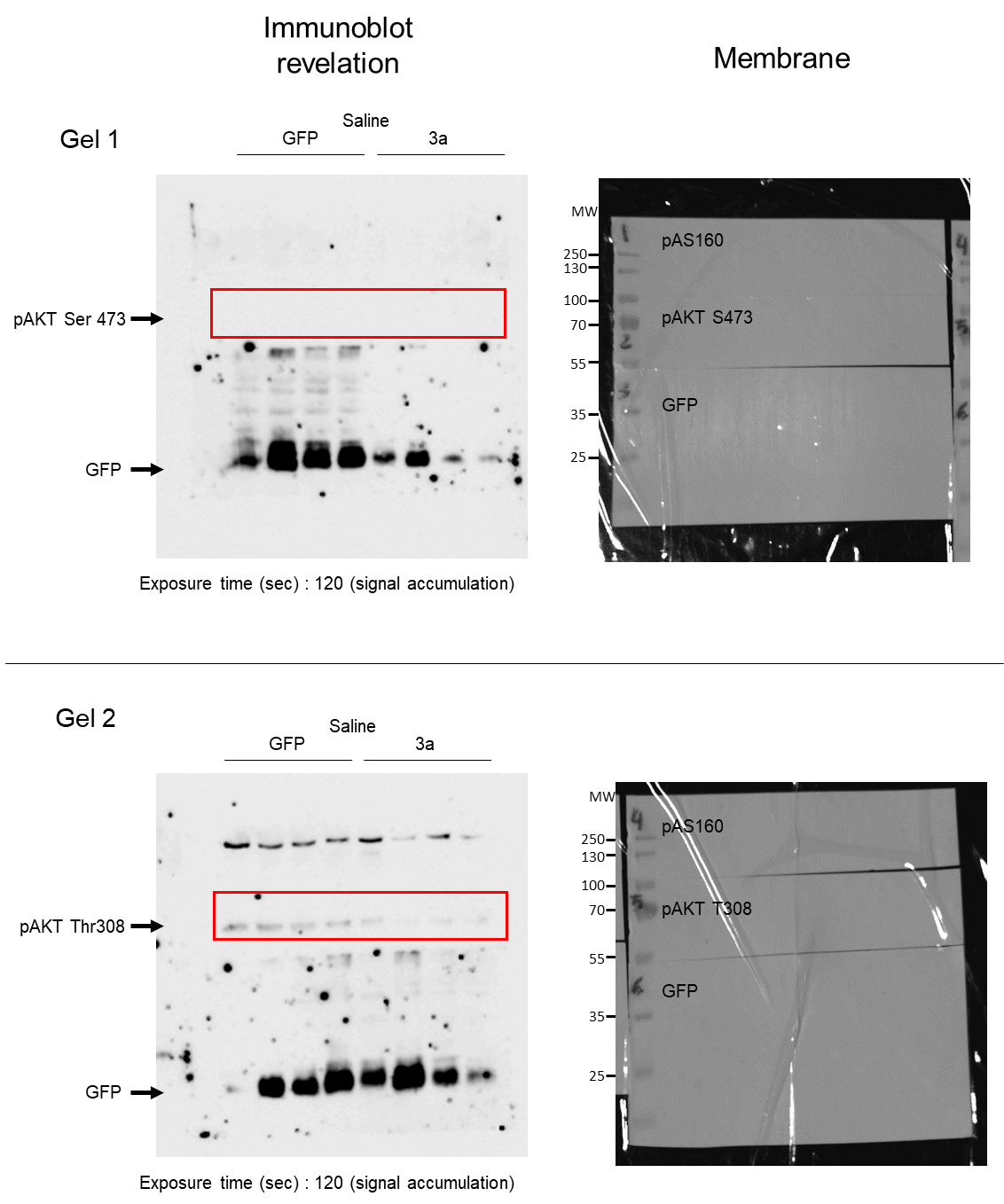


Supplementary Figure S6 Cont.

**b**


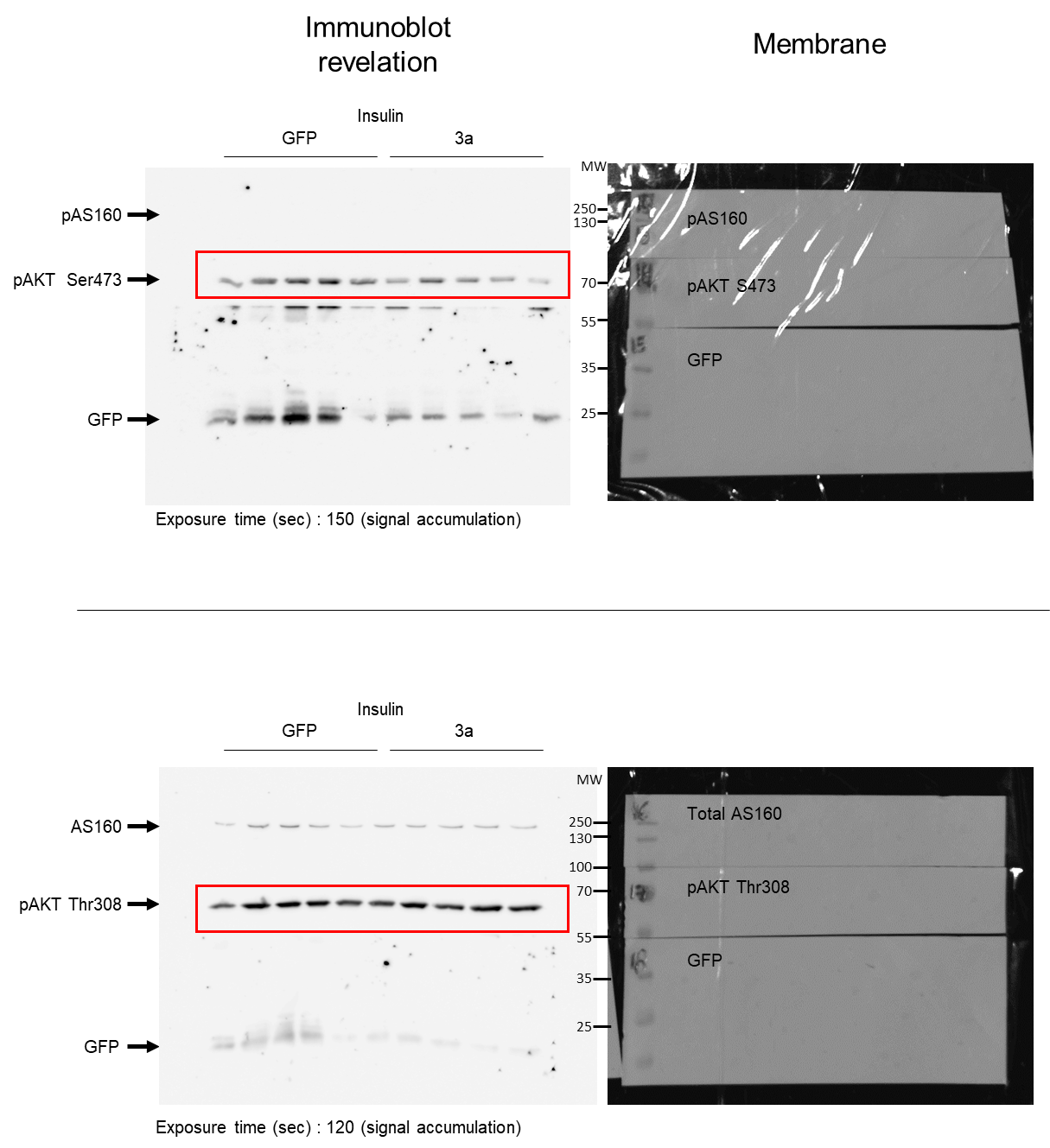


Supplementary Figure S6 Cont.

**c**


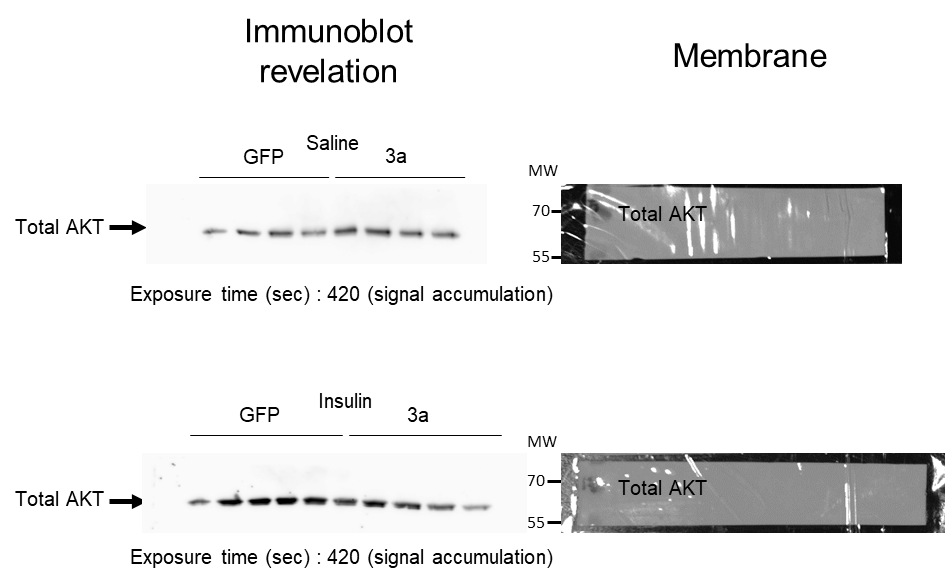


**Full immunoblots related to Figure 2d.** **(a)** Gastrocnemius samples of mice treated with saline (0.9% NaCl) were loaded on 2 separate SDS-PAGE gels for Akt signaling analysis. Membranes were cut prior blotting with the different antibodies: pAkt (Ser473 or Thr307) and GFP. GFP antibody was used to determine GFP expression levels induced by AAV8. Only the bands highlighted by a red rectangle are shown in the final figure. **(b)** Gastrocnemius samples of mice treated with insulin were loaded on 2 separate gels for Akt signaling analysis. Membranes were cut prior blotting with the different antibodies: Total AS160, pAS160, pAkt (Ser473 or Thr307) and GFP. GFP antibody was used to determine GFP expression levels induced by AAV8. Only the bands highlighted by a red rectangle are shown in the final figure. **(c)** Total Akt levels in gastrocnemius samples of mice treated with saline or insulin were assessed after stripping and reblotting of pAkt membranes with an antibody specific to total Akt.

Supplementary Figure S7


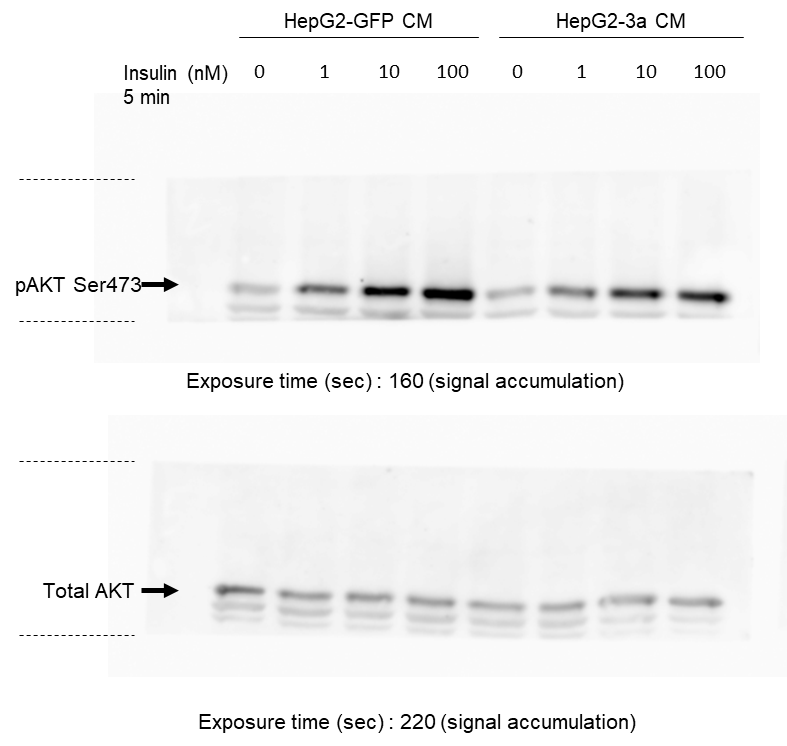


**Full immunoblots related to Figure 3b.** Lysates of C2C12 cells incubated with conditioned media (CM) from HepG2-GFP and HepG2-3a and treated with different concentrations of insulin for 5 minutes were loaded on 2 separate SDS-PAGE gels. Membranes were cut (top at 100kDa and bottom at 55kDa, the dotted lines indicate where the membranes have been cut) prior blotting with the different antibodies: Total Akt and pAkt Ser473.

Supplementary Figure S8


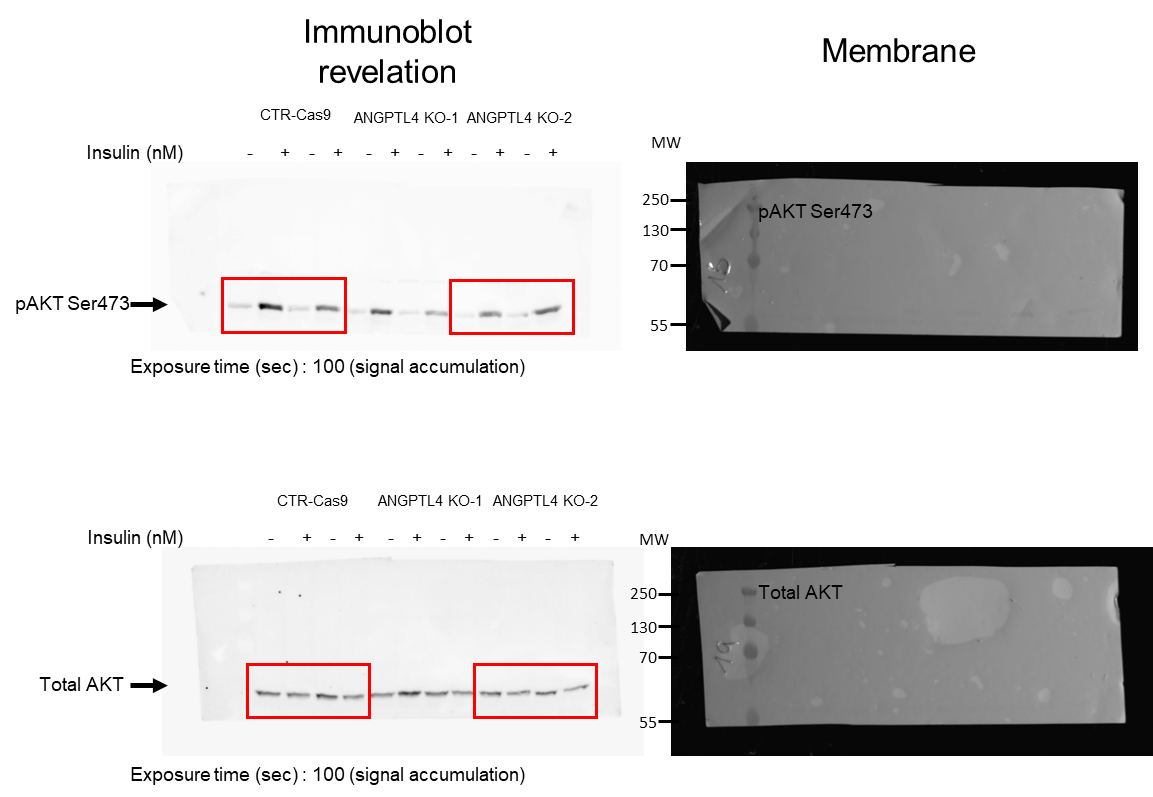


**Full immunoblots related to Figure 3e**. Samples of C2C12 cells treated with CM from non-edited (CTRL-Cas9) and edited (ANGPTL4 KO) HepG2 cells transduced with GFP or HCV-3a core were loaded on 2 separate SDS-PAGE gels. Membranes were cut prior blotting with the different antibodies: Total Akt and pAkt Ser473. Only the data of CTR-Cas9 and ANGPTL4 KO2 (guide RNA 2) were shown in the final figure as the editing with the guide RNA 1 was not efficient.

Supplementary Figure S9


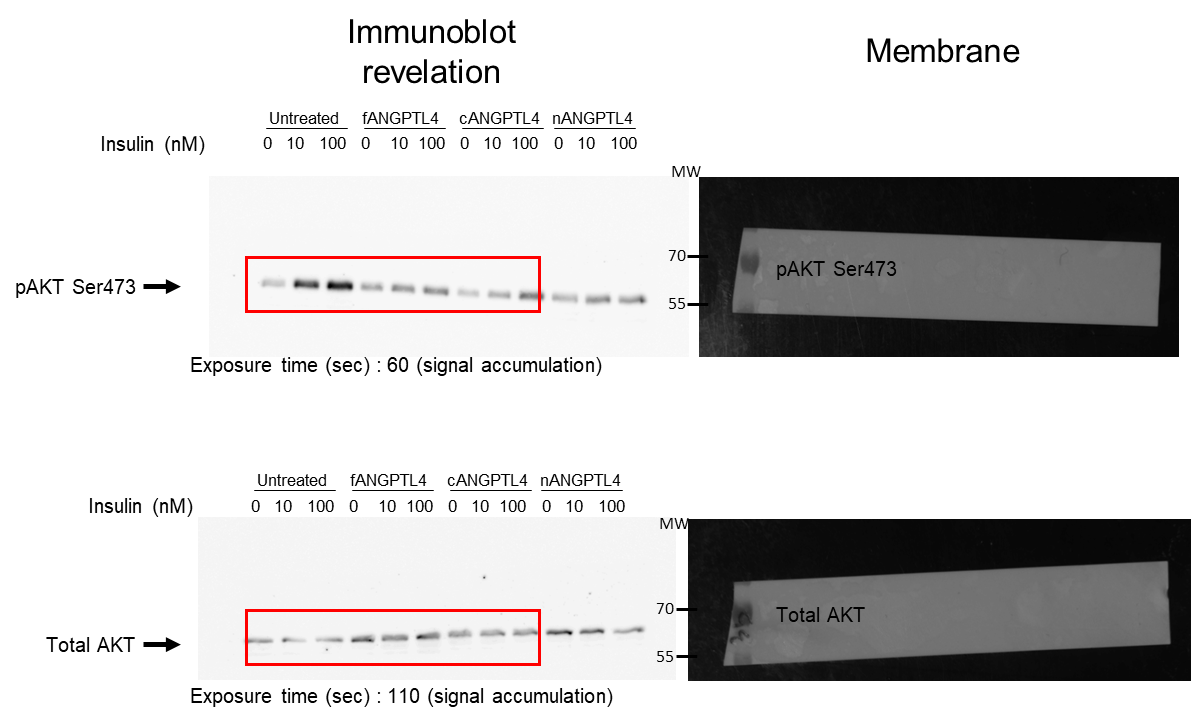


**Full immunoblots related to Figure 3f.** Lysates of human differentiated myocytes incubated with different forms of human recombinant ANGPTL4 (2.5µg/mL) and treated with different concentrations of insulin were loaded on 2 separate gels. Membranes were cut prior blotting with the different antibodies: Total Akt, pAkt Ser473. Results from nANGPTL4 were excluded from the final figure.

III- Supplementary references

1. Clement S, Pascarella S, Conzelmann S, Gonelle-Gispert C, Guilloux K, Negro F. The hepatitis C virus core protein indirectly induces alpha-smooth muscle actin expression in hepatic stellate cells via interleukin-8. J Hepatol 2010;52:635-643.

2. Sanjana NE, Shalem O, Zhang F. Improved vectors and genome-wide libraries for CRISPR screening. Nat Methods 2014;11:783-784.

3. Pietschmann T, Kaul A, Koutsoudakis G, Shavinskaya A, Kallis S, Steinmann E, Abid K, et al. Construction and characterization of infectious intragenotypic and intergenotypic hepatitis C virus chimeras. Proc Natl Acad Sci U S A 2006;103:7408-7413.

4. Scheel TK, Gottwein JM, Jensen TB, Prentoe JC, Hoegh AM, Alter HJ, Eugen-Olsen J, et al. Development of JFH1-based cell culture systems for hepatitis C virus genotype 4a and evidence for cross-genotype neutralization. Proc Natl Acad Sci U S A 2008;105:997-1002.
